# Topic Modeling of Social Networking Service Data on Occupational Accidents in Korea: Latent Dirichlet Allocation Analysis

**DOI:** 10.2196/19222

**Published:** 2020-08-13

**Authors:** Kyoung-Bok Min, Sung-Hee Song, Jin-Young Min

**Affiliations:** 1 College of Medicine Seoul National University Seoul Republic of Korea; 2 Institute of Health and Environment Seoul National University School of Public Health Seoul Republic of Korea

**Keywords:** topic modeling, occupational accident, social media, knowledge, workplace, accident, model, analysis, safety

## Abstract

**Background:**

In most industrialized societies, regulations, inspections, insurance, and legal options are established to support workers who suffer injury, disease, or death in relation to their work; in practice, these resources are imperfect or even unavailable due to workplace or employer obstruction. Thus, limitations exist to identify unmet needs in occupational safety and health information.

**Objective:**

The aim of this study was to explore hidden issues related to occupational accidents by examining social network services (SNS) data using topic modeling.

**Methods:**

Based on the results of a Google search for the phrases occupational accident, industrial accident and occupational diseases, a total of 145 websites were selected. From among these websites, we collected 15,244 documents on queries related to occupational accidents between 2002 and 2018. To transform unstructured text into structure data, natural language processing of the Korean language was conducted. We performed the latent Dirichlet allocation (LDA) as a topic model using a Python library. A time-series linear regression analysis was also conducted to identify yearly trends for the given documents.

**Results:**

The results of the LDA model showed 14 topics with 3 themes: workers’ compensation benefits (Theme 1), illicit agreements with the employer (Theme 2), and fatal and non-fatal injuries and vulnerable workers (Theme 3). Theme 1 represented the largest cluster (52.2%) of the collected documents and included keywords related to workers’ compensation (ie, company, occupational injury, insurance, accident, approval, and compensation) and keywords describing specific compensation benefits such as medical expense benefits, temporary incapacity benefits, and disability benefits. In the yearly trend, Theme 1 gradually decreased; however, other themes showed an overall increasing pattern. Certain queries (ie, musculoskeletal system, critical care, and foreign workers) showed no significant variation in the number of queries.

**Conclusions:**

We conducted LDA analysis of SNS data of occupational accident–related queries and discovered that the primary concerns of workers posting about occupational injuries and diseases were workers’ compensation benefits, fatal and non-fatal injuries, vulnerable workers, and illicit agreements with employers. While traditional systems focus mainly on quantitative monitoring of occupational accidents, qualitative aspects formulated by topic modeling from unstructured SNS queries may be valuable to address inequalities and improve occupational health and safety.

## Introduction

Occupational health and safety are fundamental components of a good work environment [1]. They are relevant not only to increased productivity but also to the moral and legal responsibilities of both employees and employers [1]. Although many strategies and programs promote occupational safety and health, fatal and nonfatal accidents in the work environment remain a global problem [1,2]. Occupational accidents include any injury, disease, or death arising through the course of employment [2]. The International Labor Organization estimates that 2.78 million workers die each year from occupational injuries, and 374 million workers suffer nonfatal work-related injuries and illnesses [2].

Occupational accident statistics vary from country to country due to differences in coverage, definitions, and classifications; data divided into fatal and nonfatal rates of occupational accidents provide similar perspectives on the risks related to occupational safety and health [2]. The paradox of low levels of nonfatal injuries but high rates of fatal accidents is observed in many countries, including South Korea [3,4]. In 2017, the rate of fatal occupational injuries reached 1.12 per 10,000 Korean workers; however, the rate of nonfatal occupational injuries was 0.54 per 100 workers [5]. This paradox may be rooted in underreporting or covering up of nonfatal occupational accidents to avoid reprisal or stigma at work or due to a perception that an injury is minor or simply part of the job [3,6]. Thus, conventional record keeping of occupational accidents may simply fail to provide the actual occupational injury rates. Furthermore, there are limitations to identifying unmet needs in occupational safety and health information, resources, and interventions for injured workers.

Due to the rapid spread of smart devices and mobile internet services, social network services (SNSs) are changing the ways in which people interact with other people who have similar interests, thoughts, services, or desires [7-9]. SNS users can also engage in knowledge sharing about specific topics (ie, academic, medical, and legal issues) [7-9]. People with work-related injuries or illnesses differ from other SNS users in how they seek professional advice and information over the internet. Employers sometimes present obstacles to injured workers or minimize their benefits, and the process of approving workers’ compensation benefits is time-consuming and frustrating. Therefore, it is inevitable that injured workers will seek legal and technical information and discuss their challenges through SNSs.

We believe that SNS data do not show the entire picture of occupational accidents but are useful for discovering hidden aspects of these accidents that are not captured by conventional surveillance. In this study, by applying topic modeling techniques to SNS data, we explored issues regarding occupational accidents that are not shared or discussed publicly but are nonetheless important.

## Methods

**Figure 1:**
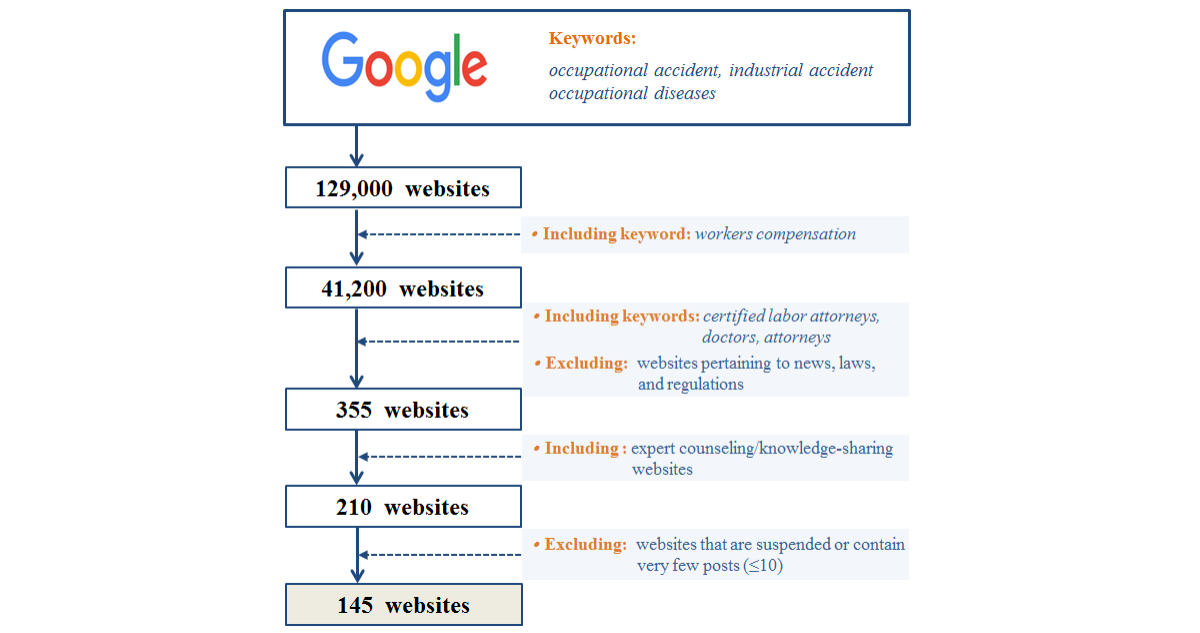
Flowchart of the identification of 145 websites (as of May 2019).

The data upon which our study is based were obtained using Google’s Korean-language search engine (Figure 1). Using the results of a Google search for the phrases *occupational accident*, *industrial accident*, and *occupational diseases*, a total of 129,000 webpages were initially selected. Next, 87,800 webpages were excluded from the initial sites by applying the keyword *workers compensation*. We further filtered the keywords *certified labor attorneys*, *doctors*, and *other attorneys*, and we excluded webpages corresponding to news, laws, and regulations. Ultimately, 355 websites were identified after applying the filter. We subsequently narrowed down our selection to 210 expert counseling and knowledge-sharing websites on which injured workers and their families could discuss difficulties related to treatment and compensation after occupational accidents and could seek answers and advice from experts. Finally, 145 websites were deemed eligible for use in the current study, excluding those that were suspended or contained fewer than 10 posts. We used web crawlers to gather website data from between 2002 and 2018; a total of 23,076 documents were collected from 145 websites. These documents were composed of full-text documents (articles or posts), comments, and blogs. From among these websites, 7832 duplicate documents were excluded. Finally, 15,244 documents were subjected to further analysis.

Prior to topic modeling analysis, the various documents were transformed into a structured form by text preprocessing. Natural language processing (NLP) was performed using KoNLPy, which is an open-source morphological analyzer for Korean processes based on the Python package [10]. Lexical analysis was performed by sentence splitting followed by tokenizing. We removed unnecessary components such as unnecessary white spaces, punctuation, special characters, and stop words such as “a,” “the,” and “it” from the unstructured data. To convert the sentences from the documents into words, tokenization was conducted using MeCab [11], an open-source morphological analyzer and part-of-speech tagger modified for the Korean language.

With keywords created through NLP, a term-document matrix was created to evaluate the importance of a word in a document. We used the term frequency–inverse document frequency (TF-IDF) technique [12], in which the term frequency measures how often a word appears in a given document divided by the total number of words while the inverse document frequency measures how frequently a word appears in all documents divided by the number of documents that contain the word. Thus, a word with a high TF-IDF score is distinctly frequent in a given document compared with other documents in the set. In this way, a term-document matrix of 35,315 words was generated from 15,244 documents. We created a co-occurrence network for high-frequency words in the given documents.

Topic modeling is a machine learning technique that is used to determine the abstract topics discussed in a given text [13]. Topic modeling is recognized as a standard methodology with high performance and convenience; it has been suggested as an alternative method of solving problems of rareness, synonyms, multiplicity, and semantic hierarchy that occur in existing word frequency analyses [13]. Latent Dirichlet allocation (LDA), a generative probabilistic model of a corpus, is a commonly used topic model. LDA assumes sparse Dirichlet prior distributions, encoding the intuition that the probability distribution of words in a topic is skewed so that only a small set of words have high probability [14,15]. In a collection of documents (D) including a word (W) and a preselected number of topics (K), LDA calculates two probabilities: the probability of words in document d assigned to topic t, P(t|d), and the probability of topic t in all the documents in a set for word w, P(w|t). The prior distributions of P(t|d) and P(w|t) are defined by the hyperparameters α and β. Gibbs sampling is used to assess distribution over topics and distribution over words for each document [13,16]. We used LDA in the Gensim library in Python for topic modeling. We set λ as 0.6 and ran the LDA with 3000 Gibbs sampling iterations. The optimal number of topics was based on perplexity and coherence (Supplemental Figure 1 in Multimedia Appendix 1). Perplexity is a method of evaluating how well a probability distribution can predict a held-out sample [17]. The smaller the change in the perplexity value, the better the probabilistic model. Topic coherence measures the score of a single topic by calculating the degree of similarity between the top N words of a topic [18]. The higher the score, the easier it is to choose the appropriate number of topics. Because there are variations in the interpretation of the quality of topics among perplexity, coherence, and human judgment [15], we further evaluated the manually formulated topics by varying the number of topics of the LDA model. Finally, we determined that 14 was a reasonable number of topics to discover hidden structures in the text body. Each topic was represented by a set of several keywords and was named by the authors of this report, who included a physician specializing in occupational medicine. These topics were categorized into three themes based on the LDA plot and the authors’ interpretation. For each topic, we performed time-series linear regression analysis with SAS statistical software (SAS Institute) by using the AUTOREG procedure to identify trends by year. The years were taken as independent variables, and the dependent variables were the average weight values for each topic by year. We classified the topics as “hot” if the regression coefficient was positive or as “cold” if the coefficient was negative, taking a 5% significance level.

## Results

Table 1 shows the numbers of documents related to occupational accidents collected on the internet by year. From the 145 websites, a total of 15,244 documents were identified during the study period (2002 to 2018). The number of documents fluctuated over time. In the first three years, from 2002 to 2005, about 100 documents (0.6% of the total documents) were identified. Occupational accident queries were very frequent in 2011 but then declined briefly. After 2012, the number of queries surged again, resulting in an average of over 1500 queries per year.

**Figure 2:**
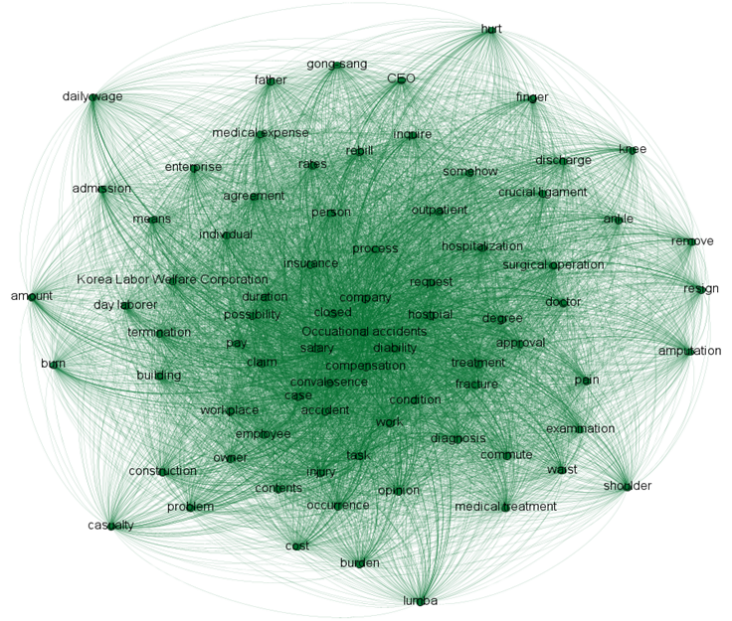
Co-occurrence network of 100 high-frequency keywords in documents related to occupational accidents.

Figure 2 shows the co-occurrence network of the high frequency keywords in the set of documents. Using keyword analysis, we investigated the degrees of connection between major keywords. The green lines indicate the connections between keywords; the darker the shade of green, the more connected the words. Among the top 100 keywords derived from TF-IDF, the keywords related to workers’ compensation showed the highest frequencies. These keywords were *company*, *process*, *salary*, *insurance*, *request*, *treatment*, and *occupational accidents.* Keywords related to occupational injuries, such as *hospitals*, *surgical operation*, *rates*, *finger*, and *hospitalization*, were also highly frequent. These high-frequency words linked to workers’ compensation and occupational injuries were interconnected with many other words.

**Figure 3:**
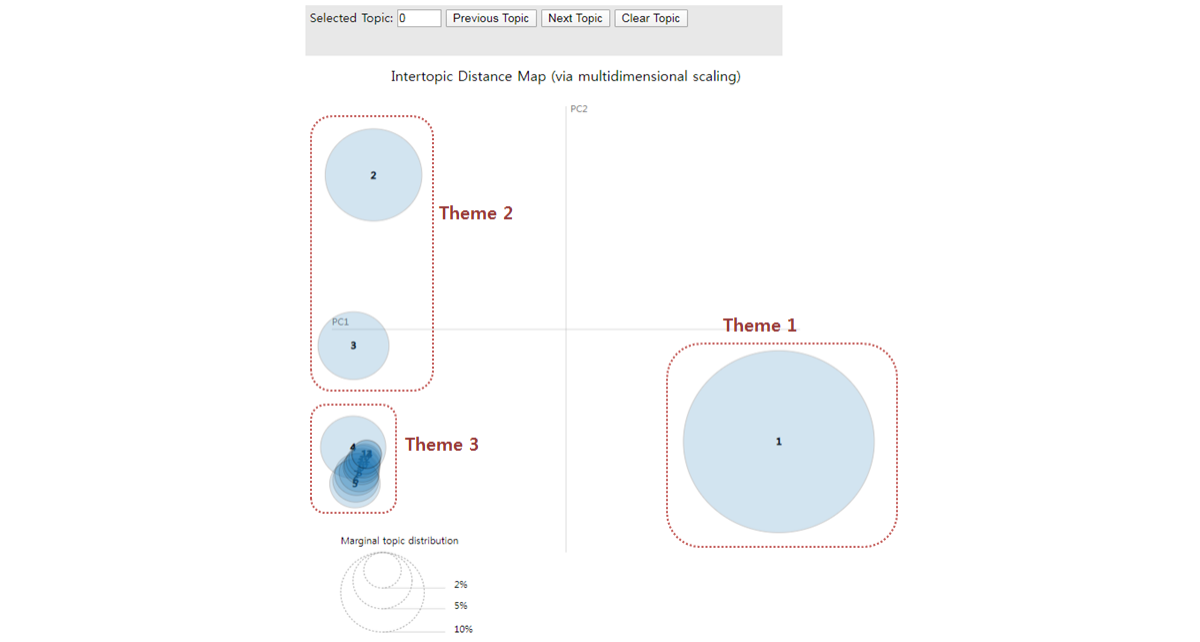
Overview of the formulation of 14 topics and 3 themes with latent Dirichlet allocation topic modeling.

Figure 3 presents the results of the LDA model; 14 topics were formulated using LDA. This set was plotted in a 2D plan along the transverse (PC1) and longitudinal (PC2) axes. Each topic was displayed as a circle, and the overall prevalence was calculated as the areas of the circles. The centers of each topic were determined by computing the distance between topics. The 14 topics were manually classified into 3 themes.

**Table 1:** Numbers of documents related to occupational accidents by year (N=15,244).

Year	Documents, n (%)
2002	9 (0.06)
2003	19 (0.12)
2004	34 (0.22)
2005	32 (0.21)
2006	104 (0.68)
2007	307 (2.01)
2008	192 (1.26)
2009	1148 (7.53)
2010	699 (4.59)
2011	480 (3.15)
2012	1602 (10.51)
2013	1977 (12.97)
2014	1774 (11.64)
2015	1754 (11.51)
2016	1508 (9.89)
2017	1934 (12.69)
2018	1671 (10.96)

Table 2 shows the formulated topics and keywords from the LDA models, including the themes, topics, subjects, keywords, and topic proportions (ie, percentages). A total of 14 topics were divided into 3 themes. The topic proportion was defined as the proportion of words in a document that belong to a topic; this measurement indicates the main topics in a document. Herein, Topic 1 appeared the most frequently, at 52.2%, and was designated Theme 1 (workers’ compensation benefits). Many of the keywords for Topic 1 were associated with workers’ compensation (ie, company, process, occupational injury, insurance, and compensation), medical expense benefits (ie, hospital, surgical operation, medical treatment, hospitalization, and convalescence), temporary incapacity benefits (ie, salary, request, approval, workplace, and shutdown), and disability benefits (ie, possibility, rates, disability, fracture, and impairment). Topics 2 and 3, which totaled approximately 20.6% of all topics, were categorized as Theme 2, illicit agreement (called gong-sang in Korean) with the employer. These two topics contained keywords related to gong-sang: specifically, related to the musculoskeletal system of Topic 2 (ie, gong-sang, disk, rupture, MRI, lumbar, traffic accident, backbone, orthopedics, surgical procedure, and X-ray) and physical trauma of Topic 3 (ie, gong-sang, thumb, metal pin, reattachment, suture, tendon, bruise, scar, stiches, and infection). The remaining Topics 4-14, approximately 27.2% of the total, included keywords describing fatal and nonfatal injuries and vulnerable workers, Theme 3. Specifically, Topics 4, 5, 6, 9, and 10 implied keywords related to fatal and nonfatal injuries, such as critical care (Topic 4), fatal accident (Topic 5), lower extremity injury (Topic 6), fracture (Topic 9), and labor-management conflict (Topic 10). The remaining topics included keywords describing vulnerable workers, such as restaurant workers (Topic 7), construction workers (Topic 8), vulnerable jobs (Topic 11), student workers (Topic 12), and foreign workers (Topic 13). In addition, Topic 14 included words such as hearing, hearing loss, hepatocirrhosis, elderly, soft tissue, sudden, and garbage man.

**Figure 4:**
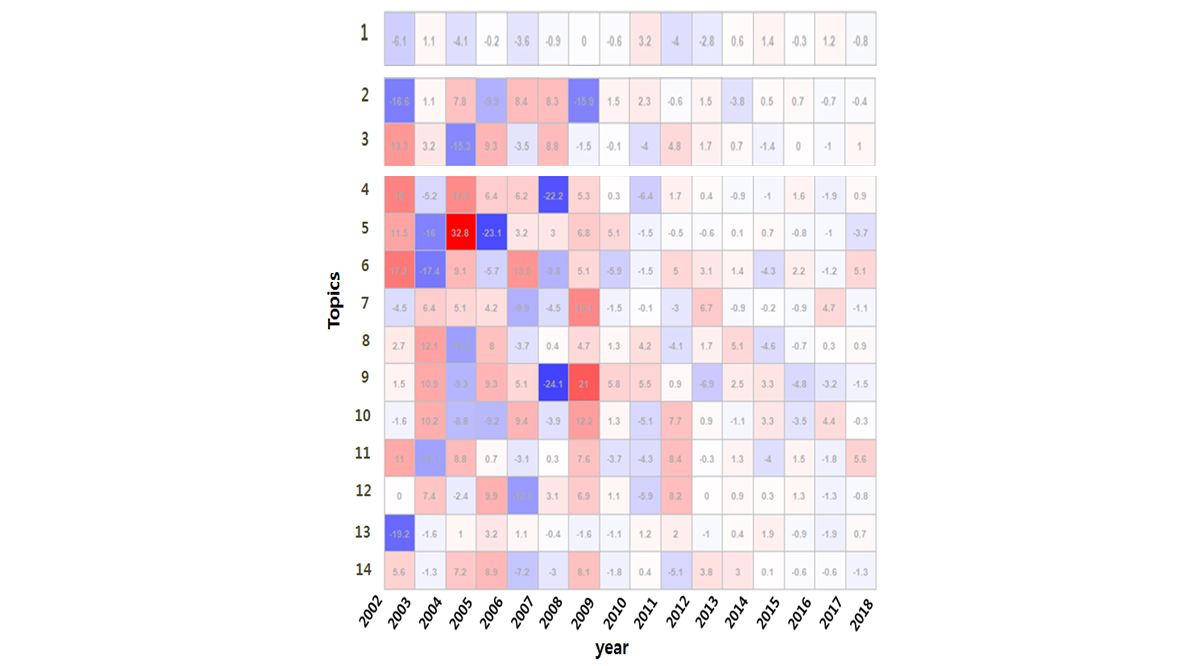
Heat map of the yearly changes in interest in the 14 topics.

Table 3 displays the regression coefficients and Figure 4 displays the heat map for the yearly changes in interest in the 14 topics from the LDA models. The largest topic was Topic 1, relating to workers’ compensation benefits; interest in this topic significantly decreased over time. In contrast, interest in Topic 2 (musculoskeletal system), Topic 4 (critical care), and Topic 13 (foreign workers) continued over time without significant changes. Most of the topics (Topic 3, Topics 5 to 12, and Topic 14) showed significant increases over time (*P*<.05).

**Table 2:** Themes, topics, and keywords formulated by latent Dirichlet allocation topic modeling.

Theme and topics		Keywords	%
**Theme 1: Workers’ compensation benefits**			52.2
	Topic 1a: Workers’ compensation	*company*, *process*, *occupational injury*, *insurance*, *compensation*	
	Topic 1b: Medical expense benefits	*hospital*, *surgical operation*, *medical treatment*, *hospitalization*, *convalescence*	
	Topic 1c: Temporary incapacity benefits	*salary*, *request*, *approval*, *workplace*, *shutdown*	
	Topic 1d: Disability benefits	*possibility*, *rates*, *disability*, *fracture*, *impairment*	
**Theme 2: Illicit agreement (*gong-sang*) with employer**			
	Topic 2: Musculoskeletal system	*gong-sang*, *disk*, *rupture*, *MRI*^a^, *lumbar*, *traffic accident*, *backbone*, *orthopedics*, *surgical procedure*, *X-ray*	13.4
	Topic 3: Physical trauma	*gong-sang*, *thumb*, *metal pin*, *reattachment*, *suture*, *tendon*, *bruise*, *scar*, *stiches*, *infection*	7.2
**Theme 3: Fatal and nonfatal injuries and vulnerable workers**			
	Topic 4: Critical care	*bleeding*, *intensive care unit*, *nothing*, *whole life*, *hospital room*, *tear*, *infection*	6.1
	Topic 5: Fatal accident	*die*, *bereaved*, *police*, *statement*, *rolled*, *autopsy*, *law*	3.7
	Topic 6: Lower extremity injury	*cast*, *myoelectric*, *numbness*, *stabbed*, *foot*, *planta pedis*, *bumped*	3.0
	Topic 7: Restaurant workers	*service*, *kitchen*, *owner*, *sick*, *piece of meat*, *hotel*, *McDonald*	2.8
	Topic 8: Construction workers	*fall*, *construction site*, *carelessness*, *Achilles tendon*, *builders*, *license*, *cement*	2.3
	Topic 9: Fracture	*open fracture*, *tumble*, *rib*, *incisura*, *cervical vertebrae*, *muscular pain*, *ventral root*	1.9
	Topic 10: Labor-management conflict	*penalty*, *headquarters*, *Labor office*, *disgusted*, *civil and criminal*, *credit*, *tape-recording*	1.8
	Topic 11: Vulnerable jobs	*construction workers*, *facilities*, *line*, *factory*, *guard*, *service industry*, *fast food*	1.7
	Topic 12: Student workers	*disadvantage*, *part-timers*, *manager*, *professor*, *college student*, *class*, *attendance*	1.4
	Topic 13: Foreign workers	*identity*, *visa*, *sojourn*, *local*, *Vietnam*, *Korea*, *reentry*	1.3
	Topic 14: Others	*hearing*, *hearing loss*, *hepatocirrhosis*, *elderly*, *soft tissue*, *sudden*, *garbage man*	1.2

^a^MRI: magnetic resonance imaging.

**Table 3:** Regression coefficients of the yearly changes in interest in the 14 topics.

Theme and topic		Estimate	*P* value	Cold/Hot
**Theme 1**				
	Topic 1: Workers’ compensation benefits	–0.2885	<.001	Cold
**Theme 2**				
	Topic 2: Musculoskeletal system	–0.0665	.10	—^a^
	Topic 3: Physical trauma	0.1194	.01	Hot
**Theme 3**				
	Topic 4: Critical care	–0.0042	.90	—
	Topic 5: Fatal accident	0.0569	.01	Hot
	Topic 6: Lower extremity injury	0.0207	.08	Hot
	Topic 7: Restaurant workers	0.0465	<.001	Hot
	Topic 8: Construction workers	0.0324	<.001	Hot
	Topic 9: Fracture	0.0178	.004	Hot
	Topic 10: Labor-management conflict	0.0261	<.001	Hot
	Topic 11: Vulnerable jobs	0.0139	.002	Hot
	Topic 12: Student workers	0.0208	<.001	Hot
	Topic 13: Foreign workers	–0.0040	.64	—
	Topic 14: Others	0.0086	.002	Hot

^a^—: no significant variation.

## Discussion

### Principal Findings

Occupational accidents are a major public health challenge [1]. Although surveillance systems have been constructed to monitor the mortality and morbidity of occupational accidents, the surveillance data may be inaccurate due to underreporting or covering up of injuries and illnesses [6,19]. This impedes proper evaluation of the magnitude of health and safety problems in the workplace, which reduces protection of workers from workplace hazards, identification of risks, and implementation of needed interventions. Our study investigated hidden issues in occupational accidents observed on the internet. We collected data from SNSs on occupational accident–related queries and analyzed them using the LDA topic model. The LDA analysis extracted a total of 14 topics, which we clustered into 3 themes: workers’ compensation benefits (Theme 1), illicit agreements with the employer (Theme 2), and fatal and nonfatal injuries and vulnerable workers (Theme 3).

The largest share of 52.2% of the collected documents concerned workers’ compensation benefits; this was classified as Theme 1. In general, if a worker is injured while working, they do not or cannot work for a certain period of time. This not only leads to the burden of medical expenses but also considerably impacts the livelihood of the worker and their family due to the suspension of household income. To ensure social security as a collective measure against injuries, disease, and death, the Korean government implemented the Industrial Accident Compensation Insurance Act 2015, in which all workers are required to participate. The Industrial Accident Compensation Insurance Act pays compensation (hereafter referred to as workers’ compensation benefits) such as medical care benefits, temporary layoff benefits, and disability benefits to workers who are injured or disabled at work [20,21]. Although Korean workers who require more than three days of treatment are covered by the Industrial Accident Compensation Insurance Act, it has been found that the approval process for workers’ compensation benefits can be protracted and difficult because insurers and employers sometimes attempt to limit injured workers’ benefits [20]. Therefore, the primary concern of workers suffering from occupational injuries or diseases may focus on obtaining compensation benefits through legal procedures rather than how the occupational injury or disease affects them. That is, affected workers need advice from legal experts (such as certified labor attorneys and other attorneys) regarding the type of compensation they are eligible for. Due to these affected workers’ needs, Theme 1 is the most frequently mentioned subject throughout the entire industrial accident query. As shown in Table 2, Theme 1 is composed of Topic 1, which includes keywords related to workers’ compensation such as *company*, *process*, *request*, *benefits*, *occupational injury*, *insurance*, *accident*, *approval*, and *compensation*. In addition, it includes keywords describing the specific benefits of industrial accident compensation, namely medical expense benefits (ie, *hospital*, *surgical operation*, *medical treatment*, *hospitalization*, and *convalescence*); temporary incapacity benefits (ie, *salary*, *request*, *approval*, *workplace*, and *shutdown*); and disability benefits (ie, *possibility*, *rates*, *disability*, *fracture*, and *impairment*).

The second-largest cluster was Theme 3, fatal and nonfatal injuries and vulnerable workers, with 28.2 percent of documents. When we considered occupational accidents and industrial accident compensation, we found that the major concern of injured or disabled workers during work was whether they could be beneficiaries of industrial accident compensation. Although numerous injuries and illnesses are known to result from occupational causes [1,22], establishing a causal relationship between work factors and injuries is a prerequisite for the approval of industrial accident compensation. Therefore, the affected workers appear to have described their occupationally related injuries on SNSs, and on the same platforms, they asked whether their accidents or illnesses would constitute legitimate industrial accidents. In Theme 3, topics 4, 5, 6, 9, 10, and 14 encompassed keywords describing fatal and nonfatal occupation-related injuries (ie, *bleeding*, *die*, *cast*, *fracture*, *incisura*, and *hearing loss*). Another issue in Theme 3 is the employment status of affected workers. Workers’ employment status affects health and causes safety disparities, which is exacerbated by unregulated and unsafe workplaces [23-25]. International reviews have found that occupational injury rates for nonstandard and temporary workers are greater than those for permanent workers [26,27]. Nonregular workers such as part-time workers, temporary contract workers, and dispatched workers may be excluded from social insurance and corporate welfare programs, and they may receive no compensation when they are affected by industrial accidents. In fact, many workers on fixed terms are not covered by workers’ compensation [28]. Keywords related to vulnerable workers (ie, *service*, *hotel*, *construction workers*, *guard*, *fast food*, and *Vietnam*) were found in topics 3, 7, 8, 11, 12, and 13 in Theme 3.

Finally, Theme 2 accounted for the smallest percentage (20.6%) of the collected documents. The subject “illicit agreement” (*gong-sang* in Korean) in Theme 2 refers to cases where injured employees negotiate settlements separately (ie, beyond the legal purview) with their employers instead of legally declaring their accidents and pursuing workers’ compensation benefits [29]. Although injuries to the musculoskeletal system (Topic 2) and physical trauma (Topic 3) were the most common types of occupational injury, they are considered to be mild injuries, and it can be difficult to prove that they are work-related. Therefore, workers’ compensation claims for these injuries are rarely approved. Thus, when workers suffer from musculoskeletal disease or physical trauma (ie, *disk*, *rupture*, *bruise*, and *reattachment*), they often enter into illicit agreements with their employers to receive payment from the companies.

We also performed a time-series analysis to investigate the annual changes in themes and topics between 2002 and 2018. Typical queries regarding the compensation for occupational accidents (seen in Theme 1) were found to decrease over time, whereas most queries related to themes 2 and 3 increased. Notably, certain queries regarding the musculoskeletal system (Topic 2 in Theme 2), critical care (Topic 4 in Theme 3), and foreign workers (Topic 13 in Theme 3) showed no significant variation in the number of queries during the study period (2002 to 2018). This appears to indicate that those topics surface consistently every year in discussions surrounding occupational accidents.

LDA topic modeling is a method that is used to identify the underlying topics contained in unstructured text data. This method is used widely in topic detection in medicine, marketing research, political science, and linguistics [16,30,31]. Our study is the first to use LDA techniques to discover latent issues related to occupational accidents. Based on web-based and unstructured web-based documents, we obtained novel insights into the unfulfilled needs of industrial accident workers and the information they sought via expert consultation as well as the occupations that are vulnerable to industrial accidents.

### Limitations

We should mention several important limitations regarding the methods used in this study. The main critique here relates to the instability of topic distribution and interpretation. Because topic modeling is sensitive to input data and analysis, changes (such as adding new documents and implementing text mining algorithms such as tokenization and stemming) can generate completely different topics. Therefore, the topics are often an amalgam, and it is difficult to assign truth to the interpretation and validation in the given corpus [32]. In addition, we focused on SNS data from experts (ie, certified labor attorneys, other attorneys, and physicians) on counseling websites between 2002 and 2018 with the aim of exploring latent issues in occupational accidents. However, depending on the SNS data source and time period collected, different topics and themes can be derived from our results. Therefore, our results may not genuinely represent the overall view of occupational accidents in our country. Moreover, applying our results to other countries with distinct industrial accident laws and regulations would require considerable attention.

### Conclusion

We conducted LDA analysis with SNS data related to occupational accidents and discovered that the primary concerns of workers posting about occupational injuries and diseases were workers’ compensation benefits, fatal and nonfatal injuries and vulnerable workers, and illicit agreements with employers. While traditional systems focus mainly on quantitative monitoring of occupational accidents, qualitative aspects formulated by topic modeling from unstructured SNS queries may be valuable for providing practical knowledge and information to the affected workers. This approach may be useful to address inequality among workers and improve occupational health and safety.

## Appendix

Multimedia Appendix 1 Supplementary table and figure.

## Abbreviations

LDA: Latent Dirichlet allocation
NLP: natural language processing
SNS: social network service
TF-IDF: term frequency–inverse document frequency
