# Supplementary material for: Topic Modeling of Social Networking Service Data on Occupational Accidents in Korea: Latent Dirichlet Allocation Analysis
Source: J Med Internet Res. 2020 Aug 13;22(8):e19222. doi: 10.2196/19222 (PMC7453332; doi:10.2196/19222)
Supplement: Multimedia Appendix 1 [file jmir_v22i8e19222_app1.docx]

| **Supplemental Table 1.** List of the 145 websites used in the study. | | | |
| --- | --- | --- | --- |
| 1 | <https://kin.naver.com/> | 37 | http://cafe.daum.net/nomu |
| 2 | http://cafe.daum.net/speedlaw | 38 | http://cafe.daum.net/hanvo |
| 3 | http://cafe.daum.net/1xxxx1 | 39 | http://cafe.daum.net/sanjaesd |
| 4 | http://cafe.daum.net/kissup0308 | 40 | http://cafe.daum.net/nodong153 |
| 5 | http://cafe.daum.net/suanmusic | 41 | http://cafe.daum.net/kim-nomusa |
| 6 | http://cafe.daum.net/samsunglabor | 42 | http://cafe.daum.net/humanlabour |
| 7 | http://cafe.daum.net/babytoto0909 | 43 | http://cafe.daum.net/cplaydp |
| 8 | http://cafe.daum.net/labor1234 | 44 | http://cafe.daum.net/labor1009 |
| 9 | http://cafe.daum.net/4dea | 45 | http://cafe.daum.net/nomu-119 |
| 10 | http://cafe.daum.net/worker119 | 46 | http://cafe.daum.net/busan49lawoffice |
| 11 | http://cafe.daum.net/labordoctor | 47 | http://cafe.daum.net/nomusa1369 |
| 12 | http://cafe.daum.net/hemorrhage | 48 | http://cafe.daum.net/youngnomusa |
| 13 | http://cafe.daum.net/sagoq | 49 | http://cafe.daum.net/nodong21 |
| 14 | http://cafe.daum.net/ta-helper | 50 | http://cafe.daum.net/taurusbj |
| 15 | http://cafe.daum.net/labordawn | 51 | http://cafe.daum.net/GLHAC |
| 16 | http://cafe.daum.net/Jedicpla | 52 | http://cafe.daum.net/bcilbannojo |
| 17 | http://cafe.daum.net/colachoe | 53 | http://cafe.daum.net/righttowork |
| 18 | http://cafe.daum.net/vnosa | 54 | http://cafe.naver.com/perfectsv |
| 19 | http://cafe.daum.net/sangae114 | 55 | http://cafe.naver.com/managers |
| 20 | http://cafe.daum.net/sanjaroad | 56 | http://cafe.naver.com/pokemonguide |
| 21 | http://cafe.daum.net/damages1 | 57 | http://cafe.naver.com/phoenice |
| 22 | http://cafe.daum.net/dasannomusa | 58 | http://cafe.naver.com/projects0 |
| 23 | http://cafe.daum.net/san-jae114 | 59 | http://cafe.naver.com/springandwinter |
| 24 | http://cafe.daum.net/sanjae1004 | 60 | http://cafe.naver.com/sonkangyong |
| 25 | http://cafe.daum.net/Lawyer-Park | 61 | http://cafe.naver.com/in4man4 |
| 26 | http://cafe.daum.net/LMSMHQS | 62 | http://cafe.naver.com/econecon |
| 27 | http://cafe.daum.net/askyourlaw | 63 | http://cafe.naver.com/cplapjc |
| 28 | http://cafe.daum.net/laborhalla | 64 | http://cafe.naver.com/epcmast |
| 29 | http://cafe.daum.net/nodongalecture | 65 | http://cafe.naver.com/boxbaram |
| 30 | http://cafe.daum.net/honamindustrial | 66 | http://cafe.naver.com/bosangmanjok |
| 31 | http://cafe.daum.net/5che-club | 67 | http://cafe.naver.com/arabee3 |
| 32 | http://cafe.daum.net/bosang77 | 68 | http://cafe.naver.com/study |
| 33 | http://cafe.daum.net/sanjae365 | 69 | http://cafe.naver.com/cybercpla |
| 34 | http://cafe.daum.net/winwin-labor | 70 | http://cafe.naver.com/rmastermega |
| 35 | http://cafe.daum.net/InsaNomu | 71 | http://cafe.naver.com/bosangmentor |
| 36 | http://cafe.daum.net/laborsos | 72 | http://cafe.naver.com/taurusbj |

| 73 | http://cafe.naver.com/seolaw | 109 | http://cafe.naver.com/ww1208 |
| --- | --- | --- | --- |
| 74 | http://cafe.naver.com/vnosa | 110 | http://cafe.naver.com/cplapeople |
| 75 | http://cafe.naver.com/labor2080 | 111 | http://cafe.naver.com/leenosa |
| 76 | http://cafe.naver.com/cpla119 | 112 | http://cafe.naver.com/nomusa33 |
| 77 | http://cafe.naver.com/hanvo | 113 | http://cafe.naver.com/newlabor |
| 78 | http://cafe.naver.com/blackclient | 114 | http://cafe.naver.com/sanjaecenter |
| 79 | http://cafe.naver.com/bestbosang | 115 | http://cafe.naver.com/lawask4972 |
| 80 | http://cafe.naver.com/laborahseong | 116 | http://cafe.naver.com/insaro |
| 81 | http://cafe.naver.com/jeunglabor | 117 | http://cafe.naver.com/antihitec |
| 82 | http://cafe.naver.com/snovelc | 118 | http://cafe.naver.com/malonomu |
| 83 | http://cafe.naver.com/insa119 | 119 | http://cafe.naver.com/bulova |
| 84 | http://cafe.naver.com/tz130507 | 120 | http://cafe.naver.com/smilelabor |
| 85 | http://cafe.naver.com/stonej | 121 | http://cafe.naver.com/yulchon1379 |
| 86 | http://cafe.naver.com/sanjaejikim | 122 | http://cafe.naver.com/0114dabosang |
| 87 | http://cafe.naver.com/nosa114 | 123 | http://cafe.naver.com/modubosang |
| 88 | http://cafe.naver.com/hana4972 | 124 | http://cafe.naver.com/sanjaenomusa |
| 89 | http://cafe.naver.com/hana4972 | 125 | http://cafe.naver.com/labor123 |
| 90 | http://cafe.naver.com/insa10004 | 126 | http://cafe.naver.com/welding468 |
| 91 | http://cafe.naver.com/nomusos | 127 | http://cafe.naver.com/bigtrust |
| 92 | http://cafe.naver.com/doctorsanjae | 128 | http://cafe.naver.com/sanjaepro |
| 93 | http://cafe.naver.com/mmsskk | 129 | http://cafe.naver.com/sanjaeconsult |
| 94 | http://cafe.naver.com/gogaphone | 130 | http://cafe.naver.com/yangnomusa |
| 95 | http://cafe.naver.com/sjnomusa | 131 | http://cafe.naver.com/glorylaborcafe |
| 96 | http://cafe.naver.com/littlewillow | 132 | http://cafe.naver.com/sanjae112 |
| 97 | http://cafe.naver.com/njtrees | 133 | http://cafe.naver.com/nicelawyer |
| 98 | http://cafe.naver.com/sagobosang | 134 | http://cafe.naver.com/shinful |
| 99 | http://cafe.naver.com/laboradviser | 135 | http://cafe.naver.com/sanjae4972 |
| 100 | http://cafe.naver.com/sanjaegood | 136 | http://cafe.naver.com/sago114 |
| 101 | http://cafe.naver.com/tvn12 | 137 | http://cafe.naver.com/in4s |
| 102 | http://cafe.naver.com/dachaum | 138 | http://cafe.naver.com/secret5505 |
| 103 | http://cafe.naver.com/jedicpla | 139 | http://cafe.naver.com/tomatosanjae |
| 104 | http://cafe.naver.com/younomusa | 140 | http://cafe.naver.com/ilsannomusa |
| 105 | http://cafe.naver.com/firstsocial | 141 | http://cafe.naver.com/supersange |
| 106 | http://cafe.naver.com/vcity | 142 | http://cafe.naver.com/sanjaekim |
| 107 | http://cafe.naver.com/linkmentor | 143 | http://cafe.naver.com/hly87 |
| 108 | http://cafe.naver.com/lifenow | 144 | http://cafe.naver.com/sanjaecafe |
|  |  | 145 | http://cafe.naver.com/nomusa114 |


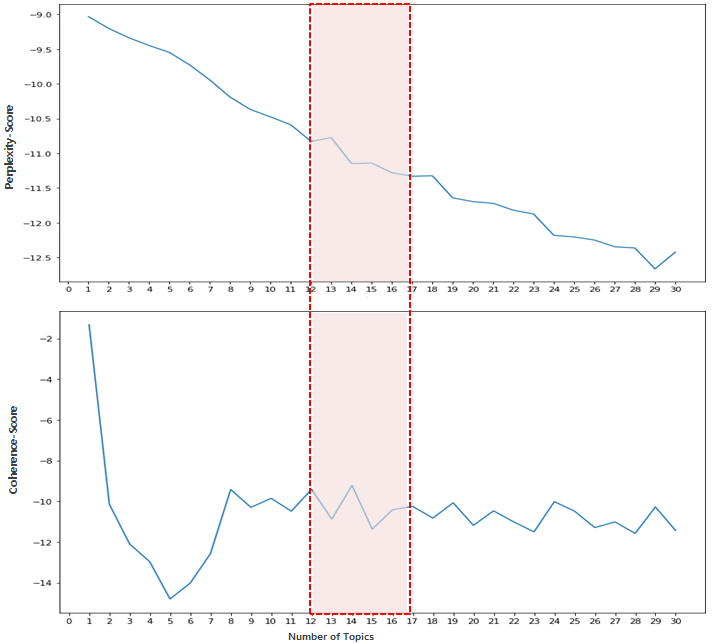


**Supplemental Figure 1.** Perplexity and coherence to determine the optimal number of topics.
